# The experience of anxiety among Chinese undergraduate nursing students in the later period of their internships: findings from a qualitative study

**DOI:** 10.1186/s12912-022-00847-9

**Published:** 2022-03-29

**Authors:** Qi-Feng Yi, Jin Yan, Cui-Jiao Zhang, Guo-Li Yang, Hui Huang, Yan Yang

**Affiliations:** 1 Nursing Department, the Third Xiang-ya Hospital of Central South University, Changsha, China; 2 Xiang-Ya Nursing School of Central South University, Changsha, China; 3 Nursing Department, the People’s Hospital of Ning-xiang, Ning-xiang, China; 4 Department of Respiratory, the Third Xiang-ya Hospital of Central South University, Changsha, China; 5 Department of Epidemiology and Health Statistics, Xiang-Ya School of Public Health, Central South University, Changsha, China; 6 Department of Cardiovascular, the Third Xiangya Hospital of Central South University, Changsha, China

**Keywords:** Anxiety, Experiences, Later internship period, Qualitative study

## Abstract

**Background:**

Clinical internships are a critical transition period for nursing students to develop into nursing professionals and are essential for the solidification of their professional attitudes. Undergraduate nursing students face multiple sources of severe anxiety that affect their nursing career development during this period. This study aimed to understand the anxiety experienced by undergraduate nursing students in the later period of their internship periods in a Chinese educational context.

**Methods:**

A descriptive qualitative study was conducted. A purposive sample of 17 undergraduate nursing interns with experiences of anxiety was selected from three teaching hospitals in Hunan Province, China**.** Data were collected through semi-structured interviews and analyzed using the Colaizzi seven-step method for data analysis.

**Results:**

Three themes emerged: the sources of perceived anxiety, the psychological journey of coping with anxiety, and suggestions for nursing management and education. The sub-themes for the first theme included career decision dilemmas, competitive pressures for employment, worries about progress of the graduation projects, challenges of the nursing licensure examination, and low self-confidence in clinical competence. The second theme consisted of two sub-themes: negative avoidance and retreat, and positive preparation and response. The third theme consisted of policy support to create more employment opportunities, comprehensive guidance from nursing schools and hospitals, and psychological assistance and counseling.

**Conclusions:**

This study revealed that undergraduate nursing students were prone to anxiety during the later period of their clinical internships. Specific sources of anxiety encompassed career decision-making, job pursuit, graduation, and licensure examination. Undergraduate nursing students adopted both positive and negative approaches in coping with anxiety during the later period of their clinical internships. Nursing educators and administrators should develop appropriate instructions and support systems to reduce anxiety among undergraduate nursing students.

## Background

A shortage of nurses affects hospital stability and quality of patient care [1]. As the backbone of the future nursing workforce, undergraduate nursing students are vital to the stability of the nursing profession and the consequent quality of care [1]. The professional values and beliefs instilled into nursing students will influence whether they choose to become nurses; nursing knowledge and skills they learned at school will affect the quality of nursing care they provide as clinical nurses after graduation [2]. Clinical internships are a crucial period for nursing students to experience clinical nursing and develop a professional identity, enabling them to transition smoothly into professional clinical nurses [3]. The later period of the internship (internship duration ≥6 months) is an important period for nursing students to decide whether to pursue a nursing career after graduation [4]; further, their mental health directly affects their lives, studies, and ultimately the stability of the future nursing workforce [5].

Undergraduate nursing education in China is a four-year program. According to the three-stage training model of public courses, professional courses, and clinical placement, students are required to complete a 10-month clinical internship in their fourth year [3]. The internship phase requires nursing students to cope with the challenges associated with their academic studies in addition to the demands of clinical practice [5]. In the later period of their internships, nursing students will have gradually adapted to the clinical work environment but are faced with multiple stressors, including concerns of employment, graduation, and licensure exams. As a result, nursing students view the internship phase as one of the most anxiety-filled periods in nursing education [5].

Anxiety is a complex emotional state involving tension, entanglement, panic, and irritability caused by confrontation with a potentially dangerous situation [6]. Certain experiences of anxiety can have positive effects on individuals; however, excessive anxiety can lead to nervousness, boredom, and sullenness; severe anxiety can even cause learning challenges and affect physical health [7].

Numerous studies have revealed that nursing students who undergo internships experience various degrees of anxiety. The reasons are multifaceted, and the main stressors might be lack of knowledge and skills, and fear of medical error resulting from inadequate preparation [8]. In addition, Chinese undergraduate nursing students need to make career decisions in the later part of their internship periods, such as deciding whether to join the nursing workforce and become clinical nurses, whether to continue their studies, or even whether to leave the nursing profession [9], which exacerbates their anxiety. The nursing licensure examination [10], operational tests, interviews, and job trials during the process of seeking jobs and thesis writing requirements [11] also add to the competitive pressure on Chinese nursing students [2, 12]. Svitsky et al. [13] revealed that the ongoing COVID-19 pandemic may also be positively related to high levels of anxiety among nursing students. It was found that perceived anxiety among nursing students in the later period of their internships was much higher than that among experienced nurses [8, 14]. A high level of anxiety may result in impaired psychological well-being and cause depression and burnout syndrome, and in extreme cases, post-traumatic stress disorder among these students, especially in the stage of clinical practice [15].

Li et al. [2, 16, 17] conducted a systematic review of synthesis interventions that promoted the mental health of nursing students. Various interventions have been found, such as psychotherapy, exercise, and training programs, among which psychotherapy showed a significant effect on decreasing students’ anxiety levels. To our knowledge, such an intervention has yet to be developed specifically for undergraduate nursing students in China. Furthermore, there are no qualitative studies examining post-practicum anxiety among nursing students in a Chinese educational context and understanding their experience of anxiety, reactions, and anticipated support during the internship period. It is critical to help students reduce their anxiety and ultimately stabilize the nursing workforce. Therefore, this study aimed to understand the anxiety experiences of undergraduate nursing students in the later period of their internships in a Chinese educational context.

## Methods

### Design

Drawing on the principles of naturalistic inquiry, a descriptive qualitative study was conducted to explore the experience of anxiety among Chinese undergraduate nursing students and strategies to alleviate anxiety during the later period of their clinical internships. Descriptive qualitative research is valuable for exploring the experiences of a phenomenon when a comprehensive summary is desired [18]. The aim of descriptive qualitative studies is to provide a descriptive summary of the desired data. Descriptive qualitative analysis is considered the method of choice when a straight description of phenomena is desired [18, 19].

### Participants and sampling method

Purposive sampling was used to recruit participants from three teaching hospitals in Hunan Province, China [20]. After obtaining the required authorization from the nursing departments of each hospital, participants were recruited by research assistants (RAs) under the supervision of the first author. The RAs were nurses working in the participating hospitals. They met the student nurses interning at the hospitals, explained the study to them, and provided them with their contact information. If students felt anxious in the clinical setting and wanted to participate in the study, they contacted RAs by phone. RAs then assessed their anxiety levels using the Self-Rating Anxiety Scale (SAS). The SAS is a reliable and validated measure of somatic symptoms associated with anxiety responses [21]. In China, in accordance with the China Mental Health Center’s recommendation [22], the standard score ranges are 25–49 (normal range), 50–59 (mild anxiety), 60–69 (moderate anxiety), and ≥ 70 (severe anxiety). The inclusion criteria for participants were: (1) undergraduate nursing interns with an internship duration of ≥6 months; (2) SAS score ≥ 50. Students who met the two inclusion criteria were invited to participate in the study. Data saturation was reached when the sample size reached 17 during the interviews. The general profile of the 17 participants, identified by Nl to N17 as their serial numbers in the chronological order of the interviews are shown in Table 1. Six of the participants were men and 11 were women. The ages of the participants ranged from 19 to 22 years (u = 20.7 years).

**Table 1:** Participant characteristics (*n* = 17)

Nursing Student No.	Genders	Suburban/Urban	Months of internship	SAS score	Graduation/ employment intention	Self-assessment of thesis progress	Self-evaluation of clinical competence
N1	F	Urban	7	53	Clinical Nursing	Dissatisfied	Satisfied
N2	M	Urban	7	55	Clinical Nursing	Average	Satisfied
N3	F	Urban	7	59	Graduate Nursing	Dissatisfied	Average
N4	F	Rural	8	63	Clinical Nursing	Average	Dissatisfied
N5	M	Rural	8	63	Clinical Nursing	Dissatisfied	Average
N6	F	Rural	8	52	Clinical Nursing	Dissatisfied	Average
N7	F	Urban	7	65	Pharmaceutical Company	Average	Dissatisfied
N8	M	Rural	7	56	Clinical Nursing	Average	Satisfied
N9	F	Rural	8	63	Clinical Nursing	Very dissatisfied	Average
N10	F	Urban	7	56	Clinical Nursing	Satisfied	Average
N11	M	Urban	8	55	Inter-professional Graduate Studies	Average	Average
N12	F	Urban	7	56	Nursing Teaching	Dissatisfied	Average
N13	F	Rural	8	66	Self-employment	Average	Very dissatisfied
N14	F	Rural	7	63	Clinical Nursing	Dissatisfied	Average
N15	M	Rural	7	60	Clinical Nursing	Average	Average
N16	F	Urban	7	68	Civil Service Examination	Very dissatisfied	Dissatisfied
N17	M	Rural	7	65	Clinical Nursing	Average	Dissatisfied

### Theoretical framework

This study is based on Jiang’s theory of “Psychological Stress System” [23], in which stressors lead to physiological, psychological, and behavioral reactions among nursing students in the later period of their clinical internships. Coping styles and social support for nursing students were considered stress-mediating variables, and anxiety was the outcome of the stress response [24]. The research interview outline was designed based on “Psychological Stress System” and is shown in Table 2. Specifically, the second question aimed to find the stressors (i.e., sources) that led to anxiety among participants; the third and fourth questions were designed to understand the coping styles participants adopted, and the social support they expected from nursing schools and hospitals.

**Table 2:** Interview guide exploring the experiences of anxiety among nursing students in the later period of their clinical internships

No.	Questions
1	How many months into your internship are you right now?
2	What is your biggest source of anxiety at the moment? What are your feelings?
3	What measures have you taken to deal with these anxieties?
4	What measures do you think schools and hospitals can implement to alleviate your anxiety?

### Data collection

The study data were collected from March 2021 to April 2021. Data were collected through face-to-face, semi-structured interviews at three teaching hospitals in the Hunan Province. Interviews were held in quiet and accessible rooms to enable participants to speak confidently. All interviews were conducted by the first author, who had 26 years of clinical training experience and was proficient in qualitative research. Participants signed the consent forms and were briefed on the purpose, procedures, and significance of the study before the face-to-face interviews. Before the interviews, the first author sought the informed consent of the participants through phone calls. Interview dates and times were also set through phone calls prior to the interviews. All participants completed a form seeking demographic information according to the instructions of the first author. The form contained questions about gender, place of residence, month of internship, graduation, or employment intention and self-assessment of thesis progress and clinical competence. All questions were open-ended except for the self-assessment portions, and were assigned a scale measuring satisfaction from 0 (very dissatisfied) to 5 (very satisfied). None of the participants dropped out during formal interviews. The researcher also tried to avoid verbal or non-verbal signs of bias to allow participants to speak freely. Interviews averaged between 20 and 30 min and were audio-recorded and then transcribed verbatim.

### Data analysis

After the interview, descriptive statistical analysis was applied to the participants’ demographic characteristics using SPSS 23.0 [25]. Two nursing professionals with qualitative research experience transcribed the audio data into text in a timely manner and compiled it into a case file. The recordings were transcribed within 24 h after the completion of the interviews. The transcribed interview data were recorded using Nvivo 11.0 software and analyzed according to Colaizzi’s [26, 27] seven-step method:

#### Step 1. Familiarization

Transcripts were reviewed numerous times to obtain a general picture of students’ perceptions and experiences of anxiety.

#### Step 2. Extraction of significant statements

We first underlined the significant statements from the transcription that could be attributed to the three interview questions (i.e., sources of perceived anxiety, psychological journey of coping with anxiety, and suggestions for nursing management and education). The questions were developed based on a psychological stress system theory [12].

#### Step 3. Formulating meanings (hidden and disclosed) from these statements

Each underlying meaning was coded and typed in the code system of Nvivo 11.0.

#### Step 4. Organization of themes into clusters

We compared the determined codes and organized formulated meanings into themes such as career decision dilemma and competitive pressure for employment which could be attributed to the “sources of perceived anxiety.”

#### Step 5. Writing an exhaustive description

In this step, the significant statements, formulated meanings, and sub-themes were integrated into exhaustive descriptions of each question.

#### Step 6. Construction of fundamental structure

We narrowed the themes and explanations of their fundamental structures. We merged similar or meaningfully similar subthemes into the main categories and reached a consensus.

#### Step 7. Returning to participants for member checking validation

A follow-up appointment was conducted between the researchers and each participant via telephone call. Each participant was given the chance to respond to the analyzed data, and alterations were made according to their feedback. Eventually, all of them agreed that the results reflected their real experiences.

### Trustworthiness

We followed Denzin and Lincoln to ensure credibility, transferability, dependability, and confirmability to verify the trustworthiness of the data [28]. The interviewer listened carefully to the thought shared by the participants during the interviews, and when there was ambiguity or any unclear statements from a participant, the interviewer repeated the participant’s statement to obtain the most accurate information. Each participant was offered a chance to correct the summary of the interview. We provided a quiet and private environment by reassuring the participants regarding the confidentiality of the information provided by them; moreover, we carried out constant reviews and detailed descriptions of how we collected data. Confirmability and constant reflection encouraged us to consider our own positionality.

## Results

Anxiety experiences included nervousness (*n* = 17), sleep disturbance (*n* = 15), unfortunate premonitions (*n* = 13), fatigue (*n* = 12), fear (*n* = 12), frequent urination (*n* = 11), excessive sweating (*n* = 10), facial flushing (*n* = 10), dizziness (*n* = 9), hand and foot tremors (*n* = 8), panic (*n* = 8), dyspnea (*n* = 8), nightmares (*n* = 6), somatic pain (*n* = 6), palpitations (*n* = 6), and stomach pain and indigestion (*n* = 6). The main themes and subthemes were categorized, as shown in Table 3. “Sources of perceived anxiety” refers to reasons which led to nursing students to perceive mild to moderate (SAS scores = 50–69) levels of anxiety. “Psychological journey of coping with anxiety” involved positive and negative methods nursing students adopted to cope with the anxiety. “Suggestions for nursing management or education” involved proposals from students regarding the potential support provided by nursing schools and hospitals to ease their levels of anxiety.

**Table 3:** Main themes and sub-themes categorized from the study

Main themes	Sub-themes
3.1 Sources of perceived anxiety	3.1.1 Career decision dilemmas
	3.1.2 Competitive pressure for employment
	3.1.3 Worries about the progress of the graduation project
	3.1. 4 Challenges of the nursing licensure examination
	3.1.5 Low confidence in clinical competence
3.1 Psychological journey of coping with anxiety	3.2.1 Negative avoidance and retreat
	3.2.2 Positive preparation and response
3.3 Suggestions for nursing management and education	3.3.1 Policy support to create more employment opportunities
	3.3.2 Comprehensive guidance from nursing school and hospital
	3.3.3 Psychological assistance and counseling

### Sources of perceived anxiety

#### Career decision dilemmas

Career decision dilemmas refers to the dilemmas faced by nursing students who were confused or unprepared to make career decisions. As nursing students approach graduation, they are faced with choices such as taking up employment or continuing their studies. Some of the interviewees chose to continue their studies in graduate school, some chose to switch to other majors for graduate studies, and others gave up their nursing majors for jobs in other fields for various reasons. Some nursing students were confused and perplexed about their career decisions and did not have clear goals.

*" I do not know whether I should choose to be an on-the-job postgraduate student or a full-time postgraduate, so I am a bit torn."—N3*

*"I did not pass the postgraduate examination this year, so I will consider holding off on employment and will continue to try again next year.”—N16*

*For many reasons, I may not choose clinical nursing, and I may not even pursue this specialty.”—N13*

Negative internship experiences also have an impact on students’ career decisions. Negative experiences include painful experiences during the internship, negative comments and actions of teachers, a perceived lack of respect for nursing staff in society, hard work on evening and night shifts, and the challenge of COVID-19.

*"I can’t stand the evening and night shifts and the job’s social status is not high. My parents want me to work in a pharmaceutical company. Moreover, I would feel upset if I gave up the nursing specialty, because I have spent four years studying this subject.”—N7*

*‘There was a shortage of protective equipment during COVID-19, and I am a little afraid that I may choose to join the civil service.”— N16*

Some nursing students have a preference for certain employment units, such as the employment units in big cities. However, the reality of the employment situation is far from their expectations, thus creating psychological pressure.

*"Although I like big provincial cities, I am afraid that I will not find a job in the provincial cities. I will hence consider returning to the local hospital for future employment as the competition there is relatively small!”—N7*

#### Competitive pressure for employment

Nursing students, especially those in the later period of their internships, felt pressure in competing with peers when looking for jobs. Seven nursing students cited anxiety due to high levels of employment stress.

*"The competition for employment is tremendous, and although there are many positions available, there are also many competitors competing with each other, and my bachelor's degree is just of the average standard in the provincial capital!"—N4*

“My parents have high expectations of me and thus, part of my concern originates from them.”—N8.

Most employers conduct several rounds of assessment before hiring, and some nursing students worry that they will not pass the recruitment exam, which triggers their anxiety.

*"‘The competitive pressure is tremendous, and I have not properly prepared for the written recruitment test, skill assessment, and interviews. Moreover, some units require a trial of practical work that makes me very nervous because operating skills are often my shortcoming.”—N13*

Recruitment information determines the amount of participation in employment opportunities, and recruitment information resources are like a lighthouse that determines the direction of individual action. Six nursing students indicated that their level of ability to actively access recruitment information resources was weak, and although they entered the career selection stage later in their internship, they still did not understand the recruitment requirements and methods, leading to anxiety.

*"Currently, I am most worried about the limited resources of recruitment channels and not knowing how to prepare for the prospective recruitment process, resume creation, etc. I was losing sleep and feeling anxiety all night.”—N4*

#### Worries about the progress of the graduation project

Undergraduate institutions require senior students to complete their research and defend their theses. Students were worried about whether they could finish the project in time for different reasons. Seven nursing students indicated that they were too busy to write their thesis because of their clinical internship, and the limited resources available to nursing students in the internship hospitals caused problems for their graduation projects.

*"I think I have difficulty coordinating between my internship and the [research] project. Currently, my graduation process is slow, and I want to finish my project by the end of May. Furthermore, I still have to defend my thesis in June, so I am really worried!”—N16*

#### Challenges of the nursing licensure examination

In April or May, before their graduation, nursing students are required to take the national licensing exam. One of the requirements for employment at many hospitals is passing the nursing licensing exam. Nursing students in the later period of their internships face challenges in preparing for the exam while simultaneously interning in hospitals and looking for jobs. Five students said that they were currently anxious about the licensure exam.

*"I am worried that I will not be able to pass the licensing exam without enough revisions, and I am worried that the hospital will not accept my application when I apply for the job."—N12*

#### Low self-confidence in clinical competence

Upon graduation and obtaining a license, nursing students become registered nurses. However, some nursing students do not have a high degree of confidence in their nursing knowledge and clinical skills; such lack of self-confidence leads to anxiety and fear. Only three nursing students rated themselves as being “confident” of their clinical competence.

*"Realizing that I am about to work alone, I feel scared and feel that my self-confidence is not enough. I believe that my operational skills need more practice, and that I should strengthen my knowledge of the theory that I have learned.”—N16*

### Psychological journey of coping with anxiety

#### Negative avoidance and retreat

Anxiety often leads to negative emotional experiences among nursing students, such as nervousness, anxiety, worry, and even fear, causing corresponding cognitive, physiological, and behavioral changes. Some nursing students reported negative effects such as feeling overwhelmed, avoidance, withdrawal, insomnia, doubts about their careers and goal shifting.

*"I am not able to sleep when I feel nervous and anxious, and sometimes I try to distract my negative emotions by shifting my attention to other areas."—N3*

*“I know that I should focus more on learning or practicing my operational skills to improve my nursing abilities, but I really cannot take action on it while anxiety is dragging me back."—N16*

#### Positive preparation and response

Moderate anxiety is protective and can improve the body’s ability to respond to environmental stimuli, which can have positive effects on work and learning. In this study, anxiety enabled some nursing students to respond actively, by paying attention to employment information, adjusting employment expectations, and preparing for employment in advance.

*"I have actively looked for information related to employment exams, such as communicating with friends, collecting information online, and preparing while studying."—N1*

*"Get help from family members to ask if there are suitable positions, read professional books to prepare, look for opportunities to apply for large hospitals, and be admitted successfully."—N8*

Some students generated positive behaviors, such as actively learning and seeking support, to successfully defend their research projects, improve their clinical competencies, and successfully pass their nursing licensure exams.

*“Advance the project as soon as possible and strive for a successful defense."—N17)*

*"Read and study for two hours a day, complete the predetermined small goals, communicate with friends, and communicate with parents to obtain support and comfort."—N7*

### Suggestions for nursing management and education

#### Policy support to provide more employment opportunities

Considering the fierce competition in finding jobs, nursing students seek policy support at the government level that could provide more employment opportunities. For example, raising wages in county and municipal hospitals, and promoting specialized training in geriatric nursing, community nursing, rehabilitation nursing, hospice care, and traditional Chinese medicine nursing.

*“Big hospitals have bigger development platforms with better benefits, so they attract people who are desperate to squeeze in. If county- and city-level hospitals can increase recruitment positions as well as improve job benefits and future career development security, I am happy to change my direction of employment."—N8*

Some nursing students also suggested the establishment of an employment platform to provide employment information. *“There is a lot of job information on the internet, but it feels like the information is scattered. A platform dedicated to providing employment information could increase our chances of getting information on employment.”—N16.*

#### Comprehensive guidance from nursing school and hospital

With respect to nursing schools, nursing students expressed their desire to have career planning education immediately after they entered the school and to receive guidance on graduation projects, on how to balance the graduation project with other commitments, on licensure exams, on employment, and on how to improve clinical competency.

*"I hope that teachers will inform students of the employment situation through continuous lectures from freshman year and by holding mock recruitment scenarios and mock job application competitions in junior year.”—N6*

*“Through meetings or information, systematically analyze the employment situation, inform students what they need to prepare for the nursing exams, how to balance subjects, nursing exams and work, so that nursing students are not so confused.”—N13*

*“I hope that the school can help broaden professional knowledge and provide skills training for skills such as first aid skills, cardiac monitoring, indwelling needle usage, and other operations to enhance professional skills."—N16*

Regarding the hospital, nursing students believed the hospital could provide more employment information in the later period of their internships by providing guidance on the employment scenario, simulations and competitions to further improve their clinical skills, and give students sufficient time to work on their graduation projects and prepare for licensure exams.

*“I hope that hospitals will provide more recruitment information in the middle and later period of the internship, so that nursing students can think more and prepare early according to the recruitment requirements and their own conditions."—N14*

*"Training on the process and form of job applications and simulating job interviews so that students can be prepared by being familiar with the form of job applications."—N4*

*“I hope the skills training room can be expanded to provide opportunities for nursing students to practice operational skills."—N16*

#### Psychological assistance and counseling

The participants mentioned that they would like clinical teachers to pay more attention to the psychological state of students, especially in the later period of their internships, and to give positive guidance and comfort to students when they ask questions or are emotionally tense. Some nursing students suggested setting up a counseling room or establishing activity groups to carry out psychological guidance activities so that nursing students can have an organization to rely on when they need it, which can effectively relieve anxiety.

*"Sometimes I feel like I need a place to breathe or someone to talk to. However, in the internship hospital, we are not familiar with each other, so I hope there could be a special psychological studio.”—N3*

Another nursing student—N13—also said, “When I feel stressed and can’t bear it alone, I hope to find a place like this.”

## Discussion

### Anxiety of nursing students in the later period of their internships deserves attention

The later period of the clinical internship is a decisive period for nursing students considering a career in nursing [4]. Our study revealed that nursing students at this stage face multiple stressors that caused anxiety, including the dilemma of making care decisions, pressure to compete for employment, worry about graduation projects and nursing licensure exams, and low self-confidence in clinical competence.

In the process of career selection, undergraduate nursing students face various psychological pressures and conflicts. For example, some nursing students desire to pursue postgraduate studies but are worried about not being shortlisted or have difficulty deciding; some nursing students wish to transfer to civil service or start their own business because they do not identify enough with nursing work or because of its physical nature, but do not want to give up their 4-year professional education. In summary, they are very confused about career decisions, and this dissonance between ideals and reality makes nursing students feel lost, confused, and anxious when choosing a career in the later period of their internships [29].

The shortage of human resources for nursing professionals is a global challenge according to the World Health Organization [13], especially challenging for countries such as China [29, 30]. The population-to-nurse ratio in China is approximately 3 per 1000, much lower than the average of more than 8 per 1000 in developed countries [31, 32]. As a reserve of nursing professionals, undergraduate nursing students are crucial to the stability of the workforce and to ensure the quality of patient care. Students affected by anxiety are at greater risk for poor academic and clinical performance, as well as decreased well-being, and this may lead to dissatisfaction with clinical practice, in turn resulting in students considering leaving the profession, thus leading to brain drain [13]. Therefore, the anxiety of nursing students in the later period of their internship deserves attention. Nursing educators and administrators should provide the necessary guidance and support, directing hospitals and academic institutions to help nursing students establish a positive career outlook and successfully complete the transition from a trainee role to a nurse’s role. This would ensure the stability and sustainable development of the nursing workforce.

### Emphasis on professional identity education

Our study found that undergraduate nursing students who were affected by anxiety in the later period of their internships exhibited poor cognizance of their professional identities and showed negative professional attitudes toward nursing. Such negative attitudes by nursing students include them considering changing majors in graduate school, changing careers, and being reluctant to engage in clinical nursing; they were even ready to give up clinical nursing work, which would result in the loss of nursing professionals.

To strengthen nursing students’ professional identities, schools can introduce ideological elements in the curriculum design and find ways to inspire nursing students and guide them in their career planning [33]. Additionally, internship hospitals can arrange nursing experts or nursing staff who have received commendations to give lectures related to professional identity by combining their personal work experience with role-model demonstrations, thereby inspiring nursing students’ professional identities.

### Post-internship targeted support and coaching

Our study revealed that nursing students need targeted training and support from the government, schools, and hospitals during the later period of their internships. For example, a sound employment service platform should be provided to undergraduate nursing students. Internship hospitals should provide employment guidance and group-based mock interviews on recruitment examination content, procedures, and skills, and help students analyze the employment scenario to improve their self-identity and mental health [9]. Interns themselves should pay attention to the integration of theory and practice, develop the ability to adapt to their environment, improve their communication skills, and continuously enhance their own professional qualities [34].

### Implementing post-internship anxiety relief strategies

In the later period of their internships, support from family, institutions, hospitals, and friends can help nursing students make a smooth transition out of nursing school [7]. As shown in our study, parents had an impact on the anxiety of nursing students. Parents should be reminded to give reasonable advice and support to nursing students to reduce anxiety through family involvement. Schools can set up student counseling stations to provide positive psychological interventions to college students free of charge, to ensure that nursing students can have a suitable channel to address and release their anxiety.

Students should also seek help from their supervising teachers for psychological guidance to relieve them from their anxiety. Meanwhile, clinical preceptors should pay attention to the psychological condition of nursing students during the post-internship period, conduct regular assessments, and provide group or individual psychological counseling to students with different personality traits, such as relaxation training, breathing training, guided reflection, positive meditation training, music therapy, and aromatherapy to help nursing students regulate their own emotions [7]. When severe anxiety symptoms last for more than 2 weeks, a visit to the psychology department is required [35].

### Implication for research and practice

Our results, combined with the literature review, suggest that nursing students in the later period of their internships faced multiple stressors that caused anxiety. Nursing educators and nursing administrators should pay attention to the factors that cause anxiety among nursing students in the later period of their clinical internships and take appropriate measures to reduce the anxiety conditions of nursing students to help them transition smoothly to nursing positions. More particularly, we recommend that nursing schools provide specific career planning courses, psychological support, and employment guidance for nursing students throughout their study. In addition, the government could set up an employment service platform that provides timely employment information. Internship hospitals should also pay attention to the psychological condition of nursing students during the post-internship period and provide psychological counseling services regularly.

### Limitations

There were some limitations to our study. First, we used a relatively small sample size with samples from only three hospitals; hence, the results may not be applicable to all nursing students at different levels and in different regional hospitals in China. Further research is necessary with students working in different institutions and regions to obtain more conclusive evidence. Another matter to consider is that the interviews were conducted in Mandarin. The verbatim data presented in this study are directly translated from Chinese, and in places the text may be difficult to understand due to cultural differences. In spite of these limitations, we believe that the study adds to the knowledge of the experience of anxiety among nursing undergraduate students in the later period of their internships and could serve as a reference for future research.

## Conclusion

This study suggests that undergraduate nursing students are prone to anxiety during the later period of their clinical internships. Specific sources of anxiety are career decision-making, job pursuit, graduation, and licensure examination. Our study revealed both positive and negative approaches adopted by undergraduate nursing students in coping with anxiety during the later period of their clinical internships, and provided specific suggestions for nursing educators and administrators in developing appropriate instructions and support systems to reduce anxiety among undergraduate nursing students.

## Data Availability

The datasets generated and/or analysed during the current study are not publicly available due to limitations of ethical approval involving the anonymity but are available from the corresponding author on reasonable request.

## Abbreviations

SAS: Self-Rating Anxiety Scale
RA: Research Assistant
